# Dynamic CCN3 expression in the murine CNS does not confer essential roles in myelination or remyelination

**DOI:** 10.1073/pnas.1922089117

**Published:** 2020-07-10

**Authors:** Nira de la Vega Gallardo, Rosana Penalva, Marie Dittmer, Michelle Naughton, John Falconer, Jill Moffat, Alerie G. de la Fuente, José R. Hombrebueno, Zhiyong Lin, Bernard Perbal, Rebecca J. Ingram, Emma Evergren, Denise C. Fitzgerald

**Affiliations:** ^a^The Wellcome-Wolfson Institute for Experimental Medicine, Queen’s University Belfast, BT9 7BL Belfast, Northern Ireland, United Kingdom;; ^b^Institute of Inflammation and Ageing, University of Birmingham, B15 2TT Birmingham, United Kingdom;; ^c^Cardiology Division, Department of Medicine, Emory University School of Medicine, Atlanta, GA 30322;; ^d^International CCN Society, 13001 Marseille, France;; ^e^The Patrick G Johnston Centre for Cancer Research, Queen’s University Belfast, BT9 7AE Belfast, Northern Ireland, United Kingdom

**Keywords:** CCN3, myelin, oligodendrocyte, OPC, remyelination

## Abstract

CCN3 is a matricellular protein that promotes oligodendrocyte progenitor cell differentiation and myelination in vitro and ex vivo. CCN3 is therefore a candidate of interest in central nervous system (CNS) myelination and remyelination, and we sought to investigate the expression and role of CCN3 during these processes. We found CCN3 to be expressed predominantly by neurons in distinct areas of the CNS, primarily the cerebral cortex, hippocampus, amygdala, suprachiasmatic nuclei, anterior olfactory nuclei, and spinal cord gray matter. CCN3 was transiently up-regulated following demyelination in the brain of cuprizone-fed mice and spinal cord lesions of mice injected with lysolecithin. However, CCN3^−/−^ mice did not exhibit significantly different numbers of oligodendroglia or differentiated oligodendrocytes in the healthy or remyelinating CNS, compared to WT controls. These results suggest that despite robust and dynamic expression in the CNS, CCN3 is not required for efficient myelination or remyelination in the murine CNS in vivo.

## Significance

Remyelination is a natural regenerative process driven by oligodendrocytes that occurs following myelin damage. Understanding this process holds therapeutic value for demyelinating diseases such as multiple sclerosis, in which remyelination can fail. CCN3 is a matricellular protein previously reported to enhance oligodendrocyte progenitor differentiation and myelination in vitro and ex vivo. Here, we show that despite extensive and dynamic expression in the murine CNS in homeostasis and following toxin-induced myelin damage, CCN3 is not required for myelination or remyelination in vivo. Yet, the anatomically distinct expression pattern suggests unidentified roles of CCN3 in a range of neurological processes. This investigation provides a framework for future investigations of the expression and role of CCN proteins in the CNS.

Myelin is a lipid-rich, lamellar structure that ensheaths axons in the peripheral and central nervous systems (CNS). In the CNS, these sheaths are formed by differentiated oligodendrocytes. Myelin enables saltatory conduction of action potentials along axons (1, 2), provides axonal metabolic support via metabolite delivery (3, 4) and is required for efficient motor skill learning (5, 6). When myelin is damaged in pathologies such as multiple sclerosis (MS), substantial neurological function deficits can arise. There are currently no therapies targeting myelin regeneration, but proof-of-concept studies and clinical trials have shown that enhancing remyelination can improve neurological function (7, 8). These reports show value in studying mechanisms that may regulate oligodendrocyte progenitor cell (OPC) differentiation and remyelination.

Recently, we reported that regulatory T cells (Treg) were necessary for efficient murine CNS remyelination (9). We also showed that Treg secrete cellular communication network factor 3 (CCN3) and that Treg-derived CCN3 enhances OPC differentiation and myelination in mixed glial and brain slice cultures, respectively (9). CCN3, previously known as nephroblastoma overexpressed (NOV) (10, 11), is a matricellular protein of the CCN family (comprising CCN1-6). CCN3 was discovered as a gene overexpressed in myeloblastosis associated virus (MAV)-induced nephroblastomas in chickens (10), and this study first reported that CCN3 is highly expressed throughout development in the brain of chickens. CCN3 has since been shown to be present in neurons in distinct areas of the rat brain (12), human fetal and adult brain, spinal cord, and in cerebrospinal fluid (13, 14). CCN3 is also expressed by primary rat astrocytes in vitro (15, 16), rat cerebral cortex astrocytes in vivo, and regulates astrocytic expression of CCL2 and CXCL1 chemokines in vitro (16). In rat brain, CCN3 was detected in Purkinje neurons and negatively regulated granule neuron precursor proliferation during cerebellar development (17). Low levels of CCN3 mRNA and protein were detected at murine embryonic development day 15 (E15) and gradually increased until postnatal day 21 (P21) (18). Interestingly, overexpressing CCN3 in mouse cerebral cortex at E14 inhibited callosal projections during development (18). These reports suggest that CCN3 plays a role in murine CNS development, but, in general, functions of CCN3 in the CNS remain poorly characterized.

Interestingly, neuronal- and Schwann cell-derived CCN2 inhibits OPC differentiation and myelination (19–21). In a model of amyotrophic lateral sclerosis, treating mice with CCN2-neutralizing antibodies decreased sciatic nerve myelin degeneration (22). CCN2 and CCN3 can mutually regulate each other as shown in chondrocyte, glomerular cell, and fibrotic disease studies (23–26) where they exhibited reciprocal negative regulation. Therefore, CCN2 and CCN3 may be a regulatory duo in OPC differentiation and myelination, providing further rationale for our central hypothesis that CCN3 supports (re)myelination in vivo.

Whether CCN3 is indeed required for efficient CNS (re)myelination remains to be determined. Furthermore, whereas *ccn3* mRNA expression in the murine CNS can be investigated in silico using in situ hybridization (ISH) and RNA sequencing (RNAseq) database resources (27–29), CCN3 protein expression in distinct anatomical regions has not been investigated. We sought to address these questions of CCN3 in myelin development and regeneration as well as provide a characterization of CCN3 expression in the adult murine CNS that will serve as a framework for future investigations of roles of CCN3 in the CNS.

## Results

### CCN3 Is Expressed by Neurons in Distinct Anatomical Regions of the Murine CNS.

*Ccn3* mRNA expression in the murine brain can be investigated in databases generated using ISH and RNAseq technologies (27–29). However, the presence of mRNA transcripts within a sample does not necessarily report the presence of corresponding protein counterparts (30, 31) and vice versa. Importantly, in many of these applications, tissue architecture is lost during processing. This is a significant limitation, particularly in the brain, which comprises many nuclei and anatomical regions, each involved in distinct neurological functions. Elucidating what cells express a protein of interest, but also where in the CNS such proteins are expressed, may help uncover CCN3 physiological functions. Furthermore, for CCN proteins (CCN2 and CCN3) there is evidence showing that in different biological systems there is no strict match between the level of CCN proteins and corresponding RNA species in various human tissues (26, 32, 33). Therefore, we sought to characterize CCN3 protein expression in the adult murine CNS using immunohistochemistry.

**Fig. 1.**
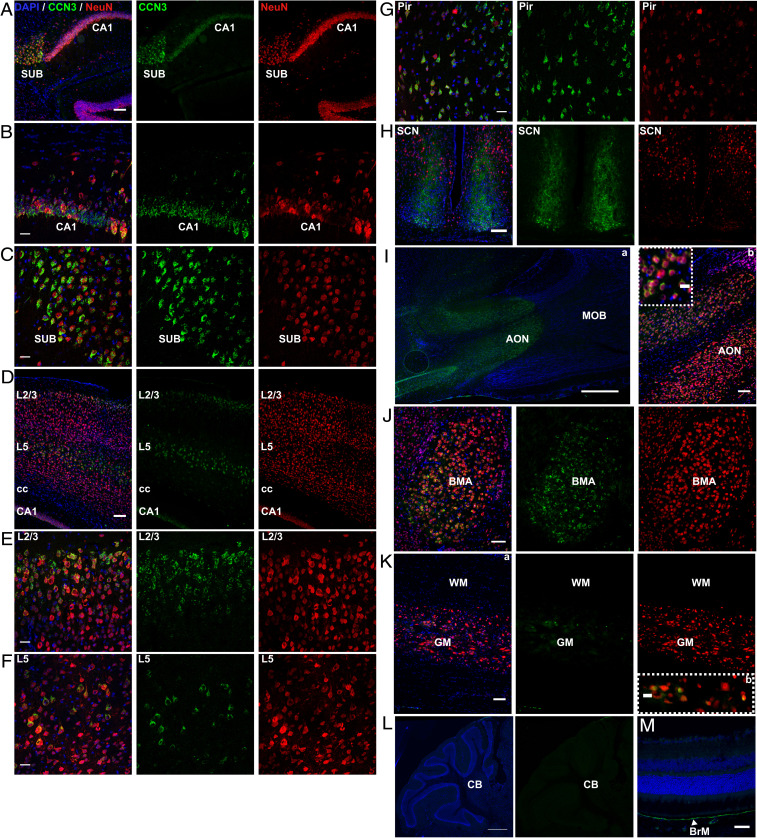
CCN3 is expressed in anatomically distinct regions of the healthy adult murine CNS. Representative images of CCN3 and NeuN staining in CA1 field of hippocampus (*A* and *B*), subiculum (*A* and *C*), cerebral cortex (*D*–*F*), piriform cortex (*G*), suprachiasmatic nuclei (*H*), anterior olfactory nuclei (*I*), basomedial amygdala nuclei (*J*), thoracic spinal cord (*K*), cerebellum (*L*), and retina (*M*). (Scale bars: 100 μm [*A*; *D*; *G*; *H*; *I*, *b*; *J*; and *K, a*], 50 μm [*M*], 25 μm [*B*; *C*; *E*; *F*; *I*, *c*; and *K*, *b*], and 0.5 mm [*I*, *a* and *L*].) AON, anterior olfactory nuclei; BMA, basomedial amygdala nuclei; BrM, Bruch’s membrane; CA1, subfield 1 of hippocampus; CB, cerebellum; cc, corpus callosum; GM, gray matter; L, layer; MOB, main olfactory bulb; Pir, piriform cortex; SCN, suprachiasmatic nuclei; SUB, subiculum; WM, white matter.

First, we validated the specificity of anti-CCN3 antibody using brain tissue from WT (*SI Appendix*, Fig. S1*A*) and CCN3^−/−^ (*SI Appendix*, Fig. S1*B*) mice and included isotype and secondary-only antibody controls (*SI Appendix*, Fig. S1 *C* and *D*). Immunohistochemical studies identified that CCN3 was expressed primarily by NeuN^+^ neurons in distinct CNS areas. A prominent site of CCN3 expression was the hippocampus, particularly the CA1 field and subiculum (Fig. 1 *A*–*C*). Interestingly, a murine cortex and hippocampal single-cell (sc) RNAseq database designates *ccn3* as a layer-specific marker of the hippocampal CA1 field (28). Likewise, using a murine scRNAseq resource (29), we found *ccn3* is the most enriched gene in excitatory neurons of the CA1 field. CCN3 was also highly expressed in the cerebral cortex, especially in layers 2/3 and 5 (Fig. 1 *D*–*F*) and piriform cortex (Fig. 1*G*). CCN3 expression was detected in every cerebral cortex anatomical region investigated (*SI Appendix*, Fig. S2 *A* and *B*).

Intriguingly, CCN3 appeared to be present extracellularly in the suprachiasmatic nuclei (SCN), suggesting CCN3 may play a role in circadian biology (Fig. 1*H*). Some CCN3^+^ cells were present in the ventrolateral SCN (Fig. 1*H*), many of which did not colocalize with NeuN. CCN3 was also detected in the anterior olfactory nuclei (AON) (Fig. 1*I*), which, together with CCN3 expression in the piriform cortex, suggests that CCN3 may play a role in olfaction. Finally, CCN3 was detected in the basomedial amygdala nuclei (Fig. 1*J* and *SI Appendix*, Fig. S2 *A* and *B*). In the thoracic spinal cord, CCN3 was detected exclusively in a few neurons of the central canal (Fig. 1*K*). CCN3 immunoreactivity was not detected in cerebellum (Fig. 1*L*), contrary to a murine ISH database showing that *ccn3 *is expressed in the Purkinje cell layer (27). Finally, CCN3 was detected in the eye in Bruch’s membrane (Fig. 1*M*), suggesting a potential role in oxygen, nutrient, or waste exchange regulation between circulation and the retina. Taken together, these results suggest that based on localization, CCN3 may have diverse roles in the CNS such as neuronal homeostasis or synaptic activity modulation in behavior, emotion, memory consolidation, and circadian regulation.

### CCN3 Is Not Required for Myelination Ex Vivo.

**Fig. 2.**
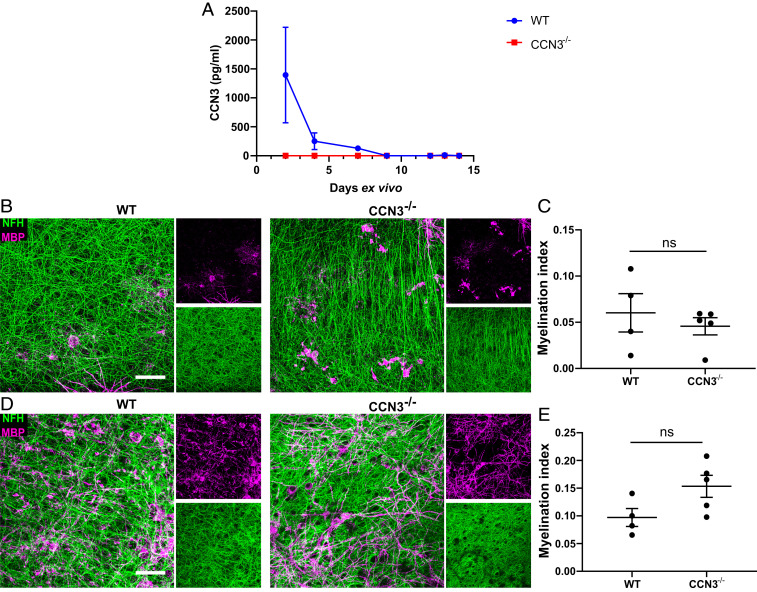
CCN3 is not required for myelination ex vivo. (*A*) CCN3 protein quantification via ELISA in cerebellar/brainstem slice conditioned media. Data are mean ± SD. Representative confocal projection images of MBP and NFH staining in brain slices at 7 (*B*) and 14 *days* ex vivo (dev) (*D*). (Scale bars: 25 μm.) Myelination index quantification (MBP^+^NFH^+^ colocalization area/NFH^+^ total area) of brain slices at 7 (*C*) and 14 dev (*E*). Data are mean ± SEM. ns, not significant. Statistical analysis: Mann–Whitney *U* test (*C*) or unpaired, two-tailed, Student’s *t* test (*E*). *n* = 3 wells containing 3 slices each (*A*) and 4–5 animals per group (*C* and *E*).

We have previously shown that exogenous Treg-derived CCN3 enhances myelination ex vivo (9). Here, we have detected that murine neonatal cerebellar/brainstem slices initially secrete CCN3. However, CCN3 becomes undetectable by day 9 (Fig. 2*A*). To investigate the cellular source of CCN3 expression in this model, WT cerebellar/brainstem slices were cultured for 2 and 4 d and immunohistochemistry was performed. Few CCN3^+^ cells were detected, and most of these cells were NeuN^+^ neurons (*SI Appendix*, Fig. S3 *B*–*F*). Whereas most of the CCN3^+^ cells were found in clusters within the brainstem, individual isolated CCN3^+^ cells were also observed (*SI Appendix*, Fig. S3 *B* and *C*). There was no significant difference in the number of CCN3^+^ or CCN3^+^NeuN^+^ cells in slices cultured for 2 or 4 d (*SI Appendix*, Fig. S3 *D*–*F*). CCN3 immunoreactivity did not colocalize with amyloid precursor protein (APP) (*SI Appendix*, Fig. S3*A*). Furthermore, the morphology of APP immunoreactivity in this tissue remained mostly cellular as opposed to bulbar.

To determine if brain slice-derived CCN3 was necessary for myelination, cerebellar/brainstem slices from WT and CCN3^−/−^ mice were cultured for up to 14 d. These slices contain resident OPCs that differentiate into myelin-producing oligodendrocytes and myelinate axons ex vivo (34). There was no difference in myelination indices between groups (MBP^+^NFH^+^ colocalization area/NFH^+^ total area) after 7 or 14 d ex vivo, suggesting CCN3 was not required for CNS myelination in this model (Fig. 2 *B*–*E*). Despite the relevance of this model, it has caveats. Brain slices are disconnected from vasculature and, therefore, from peripheral immune cells that may influence oligodendrogenesis and myelination (35) and also lack spatial and temporal regulation of myelination (36). For these reasons, as well as our findings that CCN3 is extensively expressed in the murine CNS, we sought to determine whether CCN3 was required for CNS myelination in vivo.

### CCN3 Is Not Required for Oligodendrocyte Development and Myelination In Vivo.

**Fig. 3.**
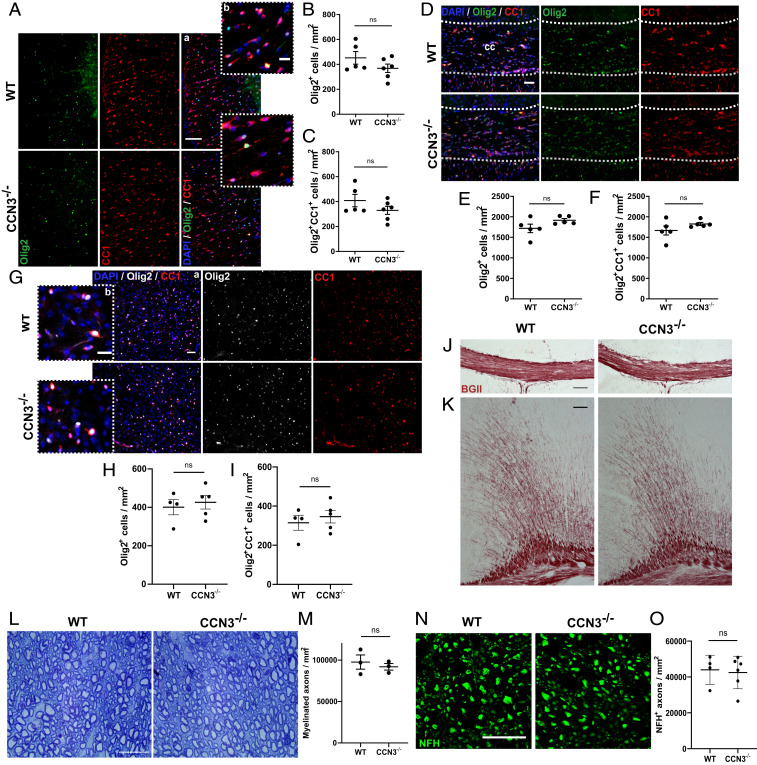
CCN3 is not required for CNS myelination in vivo. Representative images of Olig2 and CC1 staining in spinal cord vWM (*A*), medial corpus callosum (*D*), and motor cortex (*G*). OLC (*B*) and differentiated oligodendrocyte quantification (*C*) in spinal cord vWM. OLC (*E*) and differentiated oligodendrocyte quantification (*F*) in the medial corpus callosum. OLC (*H*) and differentiated oligodendrocyte quantification (*I*) in motor cortex. Representative images of Black Gold II myelin staining in medial corpus callosum (*J*) and motor cortex (*K*). (*L*) Representative images of toluidine blue staining of semithin spinal cord vWM sections. (*M*) Myelinated axon quantification in spinal cord vWM. (*N*) Representative images of NFH staining in spinal cord vWM. (*O*) Axonal quantification in spinal cord vWM. (Scale bars: 100 µm [*A*, *a*; *J*; and *K*], 50 µm [*G*, *a* and *D*], and 25 µm [*A*, *b*; *G*, *b*; *L*; and *N*].) Data are mean ± SEM. ns, not significant. Statistical analysis: (*B*, *C*, *E*, *F*, *H*, *I*, *M*, *O*) unpaired, two-tailed, Student’s *t* tests. *n* = 4–6 (*B*, *C*, *E*, *F*, *H*, *I*, and *O*) and 3 (*M*) animals per group.

To investigate if CCN3 is required for myelination in vivo, oligodendrocyte differentiation was first investigated in spinal cord and brain of healthy adult WT and CCN3^−/−^ mice. There was no significant difference in the density of Olig2^+^ oligodendrocyte lineage cells (OLCs) or Olig2^+^CC1^+^ differentiated oligodendrocytes between groups in spinal cord ventral white matter (vWM) (Fig. 3 *A*–*C*) or the corpus callosum (Fig. 3 *D*–*F*), suggesting that CCN3 is not required for oligodendrocyte development in vivo. As OLCs exhibit different morphology, proliferative capacity, and density in diverse white and gray matter (GM) regions (37), we asked whether these results were reproducible in GM. There was also no significant difference in densities of Olig2^+^ OLCs and Olig2^+^CC1^+^ oligodendrocytes between groups in the medial motor cortex (Fig. 3 *G*–*I*). Staining of myelin in CCN3^−/−^ brain tissue showed no qualitative gross deficiency in the corpus callosum or cerebral cortex (Fig. 3 *J* and *K*), suggesting CCN3 deficiency does not overtly impact CNS myelination. However, this approach does not show if myelin wrapping is impaired in the absence of CCN3. Therefore, semithin spinal cord sections from WT and CCN3^−/−^ mice were stained with toluidine blue and numbers of myelinated axons quantified in vWM. There was no significant difference in numbers of myelinated axons between groups (Fig. 3 *L* and *M*), providing further evidence that CCN3 is not required for myelination in vivo. To ensure these findings were not skewed by a difference in axon numbers between genotypes, NFH^+^ axons were quantified in vWM of WT and CCN3^−/−^ mice and no significant difference was observed (Fig. 3 *N* and *O*). Together, these results indicate that CCN3 is not essential for oligodendrocyte development or myelination in vivo.

### CCN3 Is Up-Regulated in Spinal Cord White Matter during Remyelination.

**Fig. 4.**
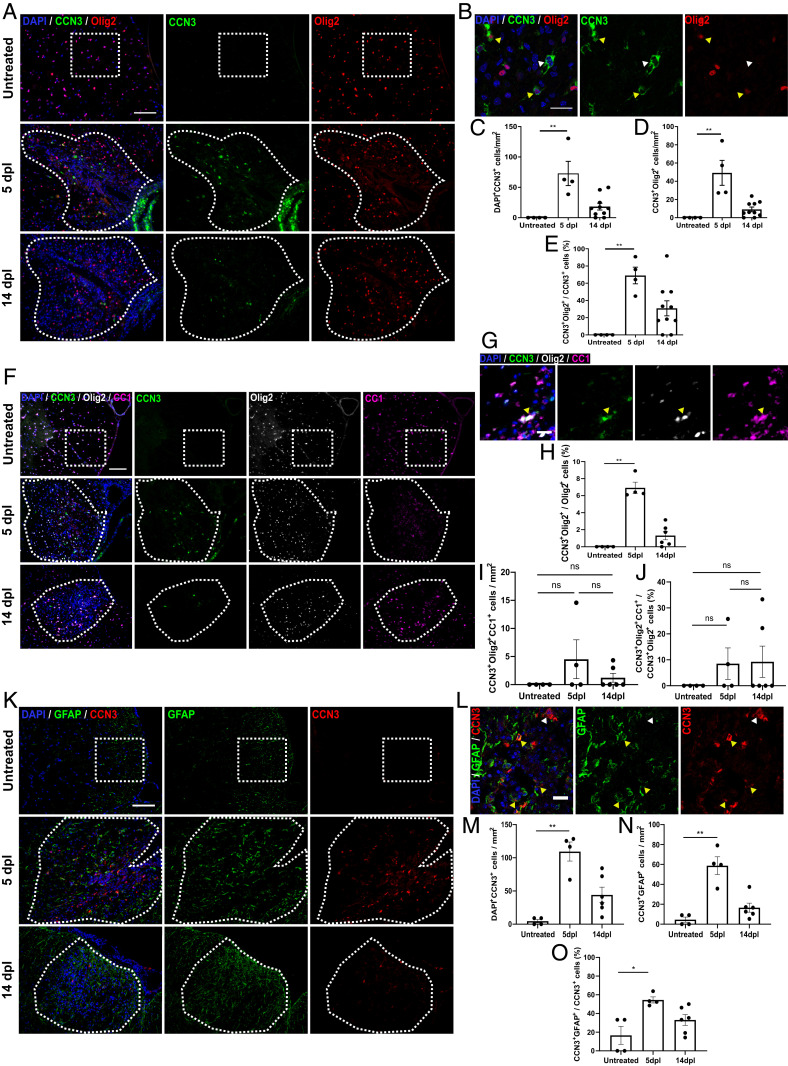
CCN3 is transiently up-regulated in the spinal cord during remyelination. Representative images of CCN3 and Olig2 (*A*), CCN3, Olig2 and CC1 (*F*), and CCN3 and GFAP (*K*) staining in spinal cord of untreated controls, and 5 and 14 dpl after lysolecithin injection. (*B*) Representative confocal images of CCN3 and Olig2 staining in spinal cord lesions at 5 dpl. White arrowheads, CCN3^+^Olig2^−^ cells; yellow arrowheads, CCN3^+^Olig2^+^ cells. CCN3^+^ cells (*C*), CCN3^+^Olig2^+^ cells (*D*), and percentage of CCN3^+^Olig2^+^ cells (*E*) quantification in untreated, 5 dpl, and 14 dpl spinal cord vWM. (*G*) Representative images of CCN3, Olig2, and CC1 staining in spinal cord lesions at 5 dpl. Yellow arrowheads, CCN3^+^Olig2^+^CC1^+^ cell. Proportion of OLCs that are CCN3^+^ (*H*), CCN3^+^Olig2^+^CC1^+^ cells (*I*) and proportion of CCN3-expressing OLCs that are CC1^+^ (*J*) quantification in untreated, 5 dpl, and 14 dpl spinal cord vWM. (*L*) Representative confocal images of CCN3 and GFAP staining in spinal cord lesions at 5 dpl. White arrowheads, CCN3^+^GFAP^−^ cells; yellow arrowheads, CCN3^+^GFAP^+^ cells. CCN3^+^ cells (*M*), CCN3^+^GFAP^+^ cells (*N*), and percentage of CCN3^+^GFAP^+^ cell quantification (*O*) in untreated, 5 dpl, and 14 dpl spinal cord vWM. (Scale bars: *F* and *K*, 100 µm; *A*, 50 µm; *B*, *G*, and *L*, 25 µm.) Data are mean ± SEM. ns, not significant; *P < 0.05; **P < 0.01. Statistical analysis: Kruskal–Wallis with Dunn’s Multiple Comparison test. *n* = 4–10 animals per group.

CCN expression is dynamically regulated in response to stress, inflammation, injury, and repair (38). To determine if CCN3 was differentially expressed during CNS remyelination, CCN3^+^ cells were quantified in demyelinating and remyelinating spinal cord lesions induced by administration of lysolecithin, at 5 and 14 d postlesioning (dpl). CCN3 expression was significantly up-regulated in lesions at 5 dpl compared to untreated controls and was still detectable at 14 dpl but at lower levels. These results suggest dynamic regulation of CCN3 expression following myelin damage and during regeneration (Fig. 4 *A*–*C*). Interestingly, ∼65% of CCN3-expressing cells were Olig2^+^ at the peak of expression (Fig. 4 *D* and *E*), showing that cells of the oligodendrocyte lineage express CCN3. These results prompted us to further investigate whether CCN3 is expressed by OPCs or differentiated oligodendrocytes in this model. Whereas most CCN3^+^Olig2^+^ cells were CC1^−^, a small number of cells were CCN3^+^Olig2^+^CC1^+^ differentiated oligodendrocytes (Fig. 4 *F*, *G*, *I*, and *J*). It is worth noting that only ∼6% of Olig2^+^ OLCs expressed CCN3 at 5 dpl, and this proportion decreased to ∼1% by 14 dpl (Fig. 4*H*). We found that CCN3 was also expressed by GFAP^+^ glia within these lesions (Fig. 4 *K*–*N*), which could be astrocytes or potentially Schwann cells (39), and that these cells constituted ∼55% of CCN3^+^ cells at 5 dpl (Fig. 4*O*). These results demonstrate that CCN3 is transiently up-regulated during spinal cord remyelination, suggest that both Olig2^+^ and GFAP^+^ glia express CCN3 during this process, and that there may be a small population of CCN3^+^ glia expressing both Olig2 and GFAP.

### CCN3 Is Up-Regulated in the Lateral Septum and Corpus Callosum during Demyelination and Concomitant Remyelination.

**Fig. 5.**
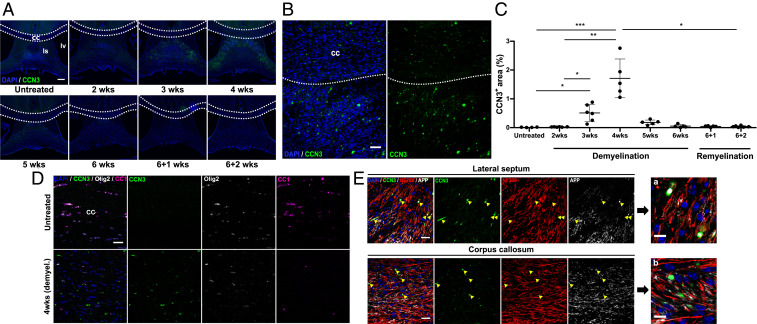
CCN3 is transiently up-regulated in the lateral septum and corpus callosum during demyelination. (*A*) Representative images of CCN3 staining in septal nuclei and corpus callosum in untreated controls, cuprizone-induced demyelination, and remyelination. cc, corpus callosum; ls, lateral septum; lv, lateral ventricle. (*B*) Representative images of CCN3 staining in the corpus callosum and lateral septum 4 wk after cuprizone-induced demyelination. (*C*) Percentage of CCN3^+^ area quantification in septal nuclei of untreated, cuprizone-fed and cuprizone-withdrawn mice. (*D*) Representative confocal images of CCN3, Olig2, and CC1 staining in the medial corpus callosum of untreated controls and WT mice fed with cuprizone for 4 wk. (*E*) Representative confocal images of CCN3, NF200, and APP staining in the lateral septum and corpus callosum 4 wk after cuprizone-induced demyelination. (Scale bars: *A*, 200 µm; *B*, 50 µm; *D* and *E*, 25 µm; *E*, *a* and *b*, 10 µm.) Data are mean ± SEM. ns, not significant; *P < 0.05; **P < 0.01; ***P < 0.001. Statistical analysis: Kruskal–Wallis with Dunn’s Multiple Comparison test. *n* = 4–6 animals per group.

To investigate if CCN3 was differentially expressed during brain demyelination/remyelination, WT mice were fed 0.2% cuprizone to induce demyelination and then changed to regular chow to allow remyelination to occur. Demyelination was confirmed by MBP, degraded MBP (dMBP) and Iba1 staining showing microglia/macrophage infiltration in the corpus callosum, the largest white matter tract in the brain and a consistent area of demyelination in this model (40) (*SI Appendix*, Fig. S1 *G* and *H*). CCN3 was detected in the corpus callosum 4 wk after cuprizone-induced demyelination (Fig. 5 *B*, *D*, and *E*). Furthermore, CCN3 was detected in the lateral septum after 3, 4, and 5 wk of demyelination and expression peaked at 4 wk (Fig. 5 *A* and *C*). To identify the source of CCN3 expression in this model, tissues were costained with cell or axonal-specific markers. CCN3 partially colocalized with NF200^+^ axons (Fig. 5*E*). Interestingly, the CCN3 immunoreactivity pattern in this tissue closely resembled that of axonal ovoids, pathologic structures which occur as a result of axonal stress, transection, or degeneration (41) and previously described during cuprizone-induced demyelination (42, 43). Moreover, CCN3 immunoreactivity partially colocalized with APP, which is expressed in neurons and accumulates in transected axons as a result of interrupted anterograde transport (44) (Fig. 5*E*). CCN3 did not colocalize with Olig2 in the corpus callosum (Fig. 5*D*), demonstrating differences in cellular sources in different models of demyelination/remyelination. This may be due to anatomical differences (spinal cord vs. brain) or the kinetics of demyelination/remyelination caused by different toxins and emphasizes the importance of comprehensive investigation of CNS remyelination. CCN3 up-regulation during CNS demyelination/remyelination led us to further investigate whether CCN3 deficiency impairs OPC differentiation during CNS remyelination.

### CCN3 Is Not Required for Efficient OPC Proliferation or Differentiation during CNS Remyelination.

**Fig. 6.**
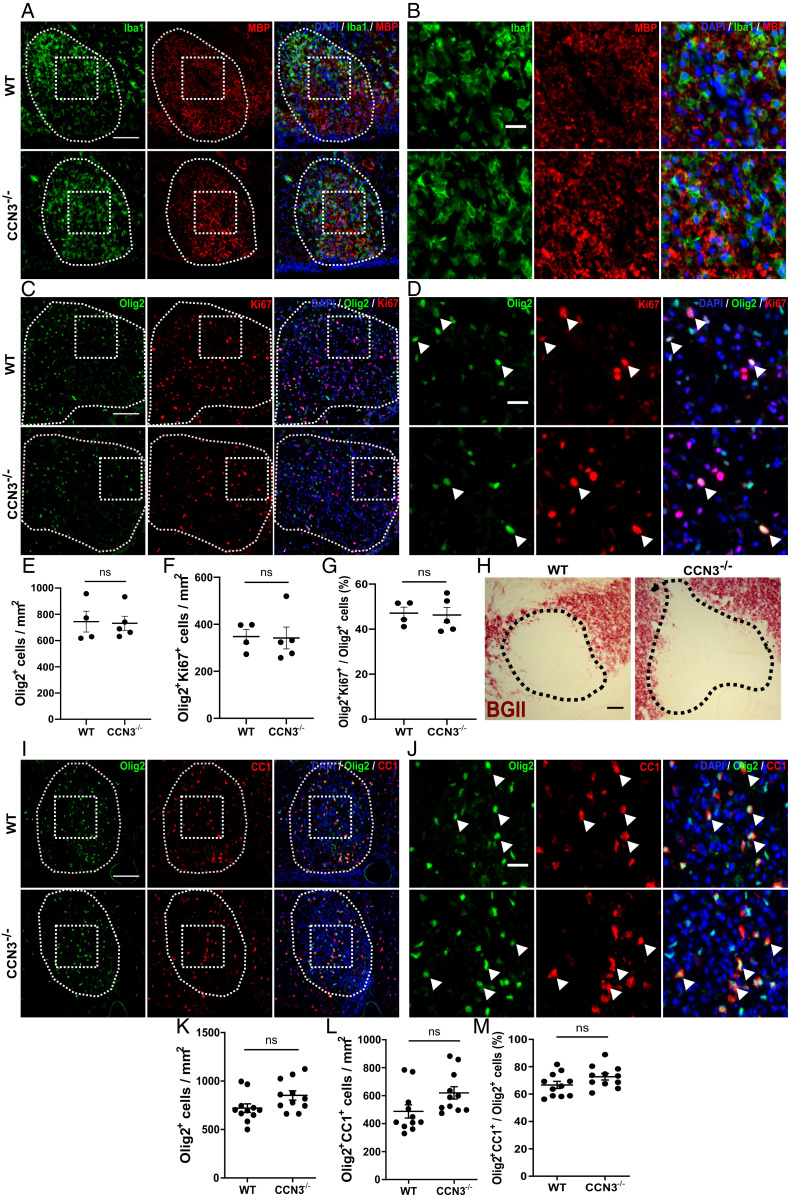
CCN3 is not required for OPC proliferation or differentiation during spinal cord remyelination. Representative images of Iba1 and MBP (*A* and *B*) and Olig2 and Ki67 (*C* and *D*) staining 5 dpl after lysolecithin injection in spinal cord vWM. White arrowheads, Olig2^+^Ki67^+^ cells. OLC (*E*), proliferating OPC (*F*), and percentage of proliferating OPC quantification (*G*) at 5 dpl. Representative images of Black Gold II (*H*) and Olig2 and CC1 (*I* and *J*) staining at 14 dpl. White arrowheads, Olig2^+^CC1^+^ cells. OLC (*K*), differentiated oligodendrocyte (*L*), and percentage of differentiated OPC quantification (*M*) at 14 dpl. (Scale bars: *A*, *C*, and *I*, 100 μm; *H*, 50 μm; *B*, *D*, and *J*, 25 μm.) Data are mean ± SEM. ns, not significant. Statistical analysis: unpaired, two-tailed, Student’s *t* tests (*E*, *F*, *K*, and *L*) or Mann–Whitney *U* tests (*G* and *M*). *n* = 4–5 (*E*–*G*) and 11 (*K–M*) animals per group.

To examine the role of CCN3 in OPC proliferation and differentiation during myelin regeneration, demyelination was induced by lysolecithin injection in the thoracic vWM of WT and CCN3^−/−^ mice. Demyelination was confirmed by MBP and Iba1 staining at 5 dpl and Black Gold II staining at 14 dpl (Fig. 6 *A*, *B*, and *H*). There was no significant difference in densities of Olig2^+^ OLCs in lesions at 5 or 14 dpl (Fig. 6 *E* and *K*), Olig2^+^Ki67^+^ proliferating OPCs at 5 dpl (Fig. 6 *C*, *D*, and *F*), or Olig2^+^CC1^+^ oligodendrocytes at 14 dpl (Fig. 6 *I*, *J*, and *L*). Likewise, there was no difference in percentages of proliferating (Fig. 6*G*) or differentiated (Fig. 6*M*) OPCs at 5 and 14 dpl, respectively.

**Fig. 7.**
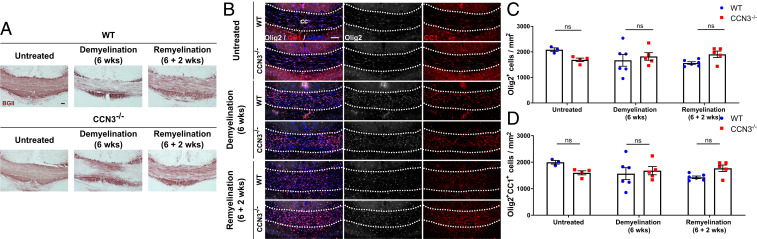
CCN3 is not required for OPC differentiation during remyelination in the brain. (*A*) Representative images of Black Gold II myelin staining in medial corpus callosum of untreated, 6 wk demyelinated and 2 wk remyelinating WT and CCN3^−/−^ mice. (*B*) Representative images of Olig2 and CC1 staining in corpus callosum of untreated, demyelinated and remyelinating mice. (Scale bars: 100 μm.) OLC (*C*) and differentiated OPC quantification (*D*) in medial corpus callosum. Data are mean ± SEM. ns, not significant. Statistical analysis: (*C* and *D*) Kruskal–Wallis with Dunn’s Multiple Comparison test. *n* = 3–6 animals per group.

To validate these results in the brain and a different model of toxin-induced demyelination, WT and CCN3^−/−^ mice were fed cuprizone to induce demyelination as described earlier and oligodendrocyte differentiation was quantified in the corpus callosum. Demyelination was confirmed by Black Gold II staining (Fig. 7*A*). There was no significant difference in the density of Olig2^+^ oligodendroglia and Olig2^+^CC1^+^ oligodendrocytes between groups (Fig. 7 *B*–*D*), suggesting that CCN3 is not required for OPC differentiation during remyelination in the CNS.

## Discussion

In this study, we provide a comprehensive characterization of CCN3 protein expression in the adult, healthy murine CNS. Using immunohistochemistry, we showed that CCN3 is expressed predominantly by NeuN^+^ neurons in specific anatomical regions of the brain and spinal cord. CCN3 was not detected in the retina or optic nerve but was detected in Bruch’s membrane. Furthermore, we show that CCN3 was transiently up-regulated during CNS demyelination and remyelination but is not necessary for efficient OPC differentiation during myelin regeneration. Likewise, healthy CCN3^−/−^ mice do not exhibit overt abnormalities in CNS OLC density or myelination. These in vivo studies are somewhat in contrast with our earlier report showing that (*i*) exogenous CCN3 purified from Treg cultures enhanced brain slice myelination ex vivo and (*ii*) depletion of CCN3 from Treg-conditioned media abrogated OPC differentiation and myelination driven by Treg-conditioned media (9). Interestingly, in pure OPC cultures, we observed that Treg-derived CCN3 did not drive OPC differentiation (*SI Appendix*, Fig. S4 *A* and *B*). This suggests the previously reported (9) positive effect of CCN3 on OPC differentiation in mixed glial cultures may have been indirect and potentially involved other CNS cell types such as astrocytes, neurons, microglia, pericytes, or even small populations of endothelial cells found within this model (45). Our findings highlight the complexity of CCN biology. Although we showed that CCN3 is expressed primarily by NeuN^+^ neurons in distinct areas of the mouse CNS, we have also shown that Olig2^+^, Olig2^+^CC1^+^, and GFAP^+^ glia express CCN3 during spinal cord remyelination. Interestingly, around 65% or 55% of CCN3^+^ cells in this model expressed Olig2 or GFAP, respectively. This discrepancy might be due to a small percentage of astrocytes and/or Schwann cells reported to arise from oligodendrocyte precursors in the remyelinating spinal cord (39, 46). Alternatively, this overlap may be due to slight variations in cell counting and/or examination of different tissue sections from the same experiment.

Regardless of the requirement (or lack thereof) of CCN3 in myelination and myelin regeneration, our CCN3 expression studies in healthy murine CNS show that CCN3 is expressed predominantly by neurons in distinct CNS regions. The brain nuclei specificity of expression suggests CCN3 may be involved in distinct functional neurological roles such as olfaction, circadian rhythm, or memory consolidation among others. Behavioral, motor function, learning and memory, stress response, and visual functional studies as well as identifying which types of neurons express CCN3 may reveal novel functions in the CNS. Importantly, our finding that CCN3 is up-regulated after CNS injury (demyelination in this case) and that it is detected in axonal ovoids characteristic of degenerating axons in the brain suggests CCN3 may be involved in neurodegeneration, opening an interesting avenue of research. An alternative explanation is that CCN3 may be produced by neurons and move along axons via axoplasmic anterograde transport, as previously suggested in ISH and immunohistochemical studies in the kidney (32). When this transport is blocked due to axonal stress or transection, CCN3 may then accumulate in axonal ovoids and become detectable. Furthermore, since CCN3 appears to be up-regulated in injury and regenerative settings, it is possible that our findings showing CCN3 detection in neurons of cerebellar/brainstem slice cultures and associated conditioned media were a result of tissue slicing during culture preparation. However, it remains a possibility that this differential expression pattern occurs as a result of developmental regulation, as opposed to injury. In summary, CCN3 is not crucial for efficient myelination or OPC differentiation during myelin regeneration in the range of murine experimental models tested, but likely plays key roles in the CNS in health and disease. These topics warrant further investigation and the studies described here lay a foundation for such studies.

## Materials and Methods

### Animals.

All mice used in this study were on a C57BL/6 background and bred in-house or purchased directly from Charles River Laboratories. Mice were housed under standard laboratory conditions: 12/12 h light/dark cycle at 21 °C and food available ad libitum. All experiments with CCN3^−/−^ mice (47), used either WT littermate controls or WT mice derived from a common original breeding line with CCN3^−/−^ mice. All animal maintenance and experiments were in compliance with the UK Home Office regulations and approved by Queen’s University Belfast Animal Welfare Ethical Review Board.

### Lysolecithin-Induced Demyelination.

Male mice between 8 and 10 wk of age were used. l-α-lysophosphatidylcholine (also known as lysolecithin) from egg yolk (Sigma) was injected in the spinal cord of mice to induce a focal demyelinated lesion as previously described (48). Briefly, 1.2 µL of a 1% (wt/vol) lysolecithin solution dissolved in phosphate buffered saline (PBS) (pH 7.4) was injected into the vWM of the thoracic spinal cord between vertebrae T12 and T13. At indicated timepoints, mice were terminally anesthetized with sodium pentobarbital and perfused transcardially with PBS followed by 4% paraformaldehyde (PFA). Spinal cords were removed, incubated overnight in 4% PFA, and cryoprotected in 30% sucrose. Spinal cords were embedded in OCT compound (Tissue Tek) and snap-frozen using 2-methylbutane (Honeywell) and dry ice. Transverse, 12-µm-thick sections were cut using a Leica cryostat. Nonconsecutive sections were collected and stained immunohistochemically for various antigens. Fluorescence and brightfield images were acquired on a Leica DM5500. Some representative images were acquired using a Leica TCS SP8 confocal microscope. Cell numbers were counted by a blinded observer in functional experiments comparing WT and CCN3^−/−^ genotypes in images of three sections per animal, and values were averaged to yield one value per animal for statistical analysis.

### Cuprizone-Induced Demyelination.

Male mice between 10 and 15 wk of age were fed 0.2% cuprizone (bis(cyclohexanone) oxaldihydrazone) (Envigo Custom Diet, TD. 140804) or normal rodent chow for untreated controls. Food and water were available ad libitum. Cuprizone feeding was maintained for up to 6 wk to induce demyelination. Where indicated, diet was then changed to normal rodent chow for an additional 2 wk to allow remyelination to occur. At indicated timepoints, mice were terminally anesthetized with sodium pentobarbital and perfused transcardially with PBS followed by cold 4% PFA. Brains were dissected, incubated overnight in 4% PFA, and cryoprotected in 30% sucrose. Brains were snap-frozen using 2-methylbutane (Honeywell) and dry ice, and coronal frozen sections of 20 µm thickness were cut using a Leica cryostat. Nonconsecutive sections between 1 mm and −1 mm relative to bregma were collected and stained immunohistochemically for various antigens. Fluorescence images were acquired on a Leica DM5500 microscope, and brightfield images were acquired on a Nikon Eclipse 80i microscope. Some representative images were acquired using a Leica TCS SP8 confocal microscope. OLC numbers were counted in the medial corpus callosum by a blinded observer in images of three to four sections per animal as above. Percentage of CCN3^+^ area was analyzed by applying a threshold in ImageJ and measuring the proportion of a delimited area with values above this threshold.

### Toluidine Blue Staining.

Male mice between 10 and 13 wk of age were perfused with 3% glutaraldehyde and 2% PFA in 0.1 M phosphate buffer (pH 7.4) with 0.7% (wt/vol) NaCl, and spinal cords were dissected and postfixed overnight. Specimens were rinsed in 0.1M cacodylate, postfixed in 1% osmium tetroxide (Agar Scientific), dehydrated, and embedded in Durcupan resin (Sigma). Semithin sections (750 nm) were cut on a Leica UCT ultramicrotome using a Histo knife (Diatome) and mounted on microscope slides. Semithin sections were incubated in a 1% toluidine blue solution for 43 s at 60 °C and rinsed with water. Brightfield images of healthy vWM tissue derived from lesioned spinal cords were acquired on a Leica DM5500 microscope. The number of myelinated axons were counted by a blinded observer on three to four sections per animal as above.

### Black Gold II Myelin Staining.

Frozen brain and spinal cord sections were stained with Black Gold II (BGII) (AG105, Millipore) according to manufacturer’s instructions. In brief, sections were washed in water, immersed in BGII solution at 60 °C, and incubated for 20 min, followed by incubation in 1% sodium thiosulfate at 60 °C for 5 min. Slides were rinsed in water, dehydrated, incubated in Clearene (Leica) for 2 min, and coverslipped using DPX mounting media (Sigma). Images of brain and spinal cord sections were acquired using DM5500 and Nikon Eclipse 80i microscopes.

### Immunofluorescence Staining of CNS Tissue.

Frozen sections were blocked with 10% normal donkey (Sigma) or goat serum (NGS) (Vector Laboratories) and incubated with primary antibodies for various antigens (*SI Appendix*, Table S1) overnight at 4 °C. When staining with primary antibodies against murine CCN3, an isotype control (catalog no. I-5000, Vector Laboratories) was used at the same final concentration. Tissue sections were then treated with secondary antibodies and incubated for 1 h at room temperature (RT) (*SI Appendix*, Table S1). For CC1 and Ki67 staining, antigen retrieval using 10% citrate buffer (Sigma) was performed. A mouse-on-mouse detection kit was used before CC1 and NF200 staining (Vector Laboratories). Cell nuclei were stained using 4′,6-diamidino-2-phenylindole, dihydrochloride (DAPI) (Thermo Fisher Scientific) before coverslipping with ProLong Gold Antifade (Life Technologies). Images of brain and spinal cord sections were acquired on Leica DM5500, TCS SP5 confocal, or TCS SP8 confocal microscopes. Some microscopy images were stitched using the “Stitching” plugin in Fiji (Fig. 1*I*) (49) or the Leica LAS X Navigator (*SI Appendix*, Figs. S2 *A* and *B* and S3 *B* and *C*).

### Characterization of CCN3 Expression in the CNS.

Male WT mice between 12 and 23 wk of age were perfused with PBS followed by cold 4% PFA between 1300 and 1900 hours. Brains, thoracic spinal cords, and eyes (with attached optic nerve) were dissected and processed as previously described. Coronal and sagittal brain frozen sections of 20 µm thickness were cut using a Leica cryostat. Nonconsecutive coronal sections between 1 mm and −2 mm relative to bregma were collected, and nonconsecutive sagittal sections of one hemisphere per brain were collected. Spinal cord (transverse and longitudinal) and eye sections of 12-µm thickness were cut using a Leica cryostat. After sections were mounted on microscope slides, they were stained immunohistochemically for various antigens and imaged as described above. Qualitative assessment of CCN3 expression was performed using at least five animals.

### T Cell Culture.

CD4^+^ T cells were isolated using an EasySep negative selection kit (19852, Stem Cell Technologies) from splenocytes of male and female, 6–14 wk old, WT mice. Isolated cells consisted of >90% CD4^+^ T cells. T cells were activated using plate-bound anti-CD3 (1 µg/mL, clone 145–2C11) and soluble anti-CD28 (1 µg/mL, clone 37.51) (both eBioscience) for 72 h in Treg-polarizing conditions: rhTGF-β (4 ng/mL, R&D Systems), rhIL-2 (20 ng/mL, eBioscience), and anti-IFN-γ (10 µg/mL, clone XMG1.2, Bioxcell). Culture media consisted of RPMI 1640 (Life Technologies) supplemented with 10% fetal calf serum (FCS), 1% penicillin/streptomycin, 1% l-glutamine, 1% Hepes, 1% sodium pyruvate, 1% nonessential amino acids, and 50 nM β-mercaptoethanol (all Life Technologies). T cells were reactivated in brain slice medium (described below) for another 72 h, and conditioned media (CM) were collected. T cell polarization was verified by flow cytometry, and Treg cultures consisted of >80% Foxp3^+^ T cells.

### CCN3 Quantification and Depletion.

Concentration of murine CCN3 in media from Treg and brain slice cultures was quantified by ELISA (DY1976, R&D Systems) according to manufacturer instructions. Results were calculated using linear regression analysis. Values plotted as 0 were below the detection limit of the kit. CCN3 was depleted from Treg-CM using monoclonal anti-CCN3 antibody-coupled magnetic beads (clone 231216, R&D Systems and Thermo Fisher Scientific) or isotype-coupled beads (clone 54447, R&D Systems). Antibody-bead coupling was performed according to manufacturer’s instructions. Briefly, 1 mg of beads was incubated with 500 µg/mL antibody. To immunoprecipitate CCN3, up to 1 mL of Treg-CM was added to 1 mg of antibody-coupled magnetic beads and incubated for 2 h on a rotator at RT, vortexing the samples every 15 min. Samples were then placed in a magnetic stand, and CCN3-depleted Treg-CM and isotype-treated controls were collected.

### OPC Isolation and Culture.

Pure OPC (POPC) cultures were generated from male and female WT P5-9 mice as previously described (45). At day 9 in culture, PDGFαα and NeuroTrophin 3 (NT3) were withdrawn to allow oligodendrocyte differentiation and cells were stimulated with 5% Treg-CM, 5% CCN3-depleted Treg-CM, 5% IgG isotype-depleted Treg-CM, or controls for up to 3 d. Purity was assessed in random cultures at 3–5 d after setup and was over 93%.

POPCs were fixed directly in 4% PFA (pH 7.4) (Sigma) for 15 min at RT. POPCs were blocked in 10% NGS (Vector Laboratories s-1000) with 0.1% Triton X-100 in PBS for 1 h. Cells were then incubated with primary antibodies for Olig2 (1:400 Millipore AB9610) and MBP (1:1,000 Millipore MAB386) in 2% NGS with 0.01% Triton X-100 in PBS overnight at 4 °C. Afterward, OPCs were incubated with secondary antibodies (1:1,000, goat anti-rabbit 488, A11034 and 1:1,000, goat anti-rat 594, A11007, both Life Technologies) for 1 h at RT. Cells were stained with DAPI for 5 min at RT. Immunofluorescence was detected using an EVOS FL microscope at 10× magnification and *n* = 5 wells per condition.

### Cerebellar/Brainstem Slice Culture.

Cerebellar/brainstem slices (300 µm) from male and female WT and CCN3^−/−^ P0–2 mice were prepared using a McIlwain Tissue Chopper and cultured in transwell inserts with brain slice medium consisting of 46.6% minimum essential medium (Thermo Fisher Scientific), 25% Earle’s balanced salt solution (Sigma), 25% heat inactivated horse serum (HIHS) (Thermo Fisher Scientific), 1% penicillin/streptomycin Thermo Fisher Scientific), 1% glutamax (Life Technologies) and 1.4% d-(+)-glucose solution (G8769, Sigma). Slices of WT or CCN3^−/−^ mice were prepared on separate days and age matched. Slices were allowed to myelinate for up to 14 d. After culture, slices were fixed in 4% PFA for 45 min at RT, washed in PBS, and blocked/permeabilized for 3 h in solution containing 2% HIHS, 10% NGS, 1% BSA (Melford Laboratories), and 0.25% Triton X-100 (Sigma). Slices were stained for MBP (1:600, clone 12, Millipore) and axonal NFH (1:250–1:400, polyclonal, EnCor Biotechnology) for 2 d overnight at 4 °C. Slices were then washed three times for 1 h each in 0.01% Triton X-100 in PBS and incubated with secondary antibodies (1:500, goat anti-rat 568, AF11077 and 1:500, goat anti-chicken 488, AF11039, both Thermo Fisher Scientific) overnight at 4 °C. Slices were stained with DAPI for 10 min at RT, mounted with ProLong Gold Antifade (Life Technologies), and imaged with a Leica TCS SP8 confocal microscope at 0.5-µm intervals. The whole slice thickness was imaged. Three fields of view (FOV) were imaged per slice, and three slices were imaged in this manner per animal (total of nine FOV per animal were analyzed to generate one mean value per animal for statistical analysis). An ImageJ macro (34) was used to determine the myelination index (MBP^+^NFH^+^ colocalization area/NFH^+^ total area). Only images within the z-stack with uniform NFH staining were included in the analyses. To quantify the number of CCN3^+^ cells in cerebellar/brainstem slices, slices were stained for CCN3, NFH, and NeuN with primary antibody concentrations indicated in *SI Appendix*, Table S1 and a 1:500 dilution factor for corresponding secondary antibodies. One focal plane overview of every slice was imaged using Leica LAS X Navigator software and a TCS SP8 confocal microscope. The number of cells were then counted using Fiji software. A total of three slices were imaged in this manner per animal to generate one mean value per animal for statistical analysis.

### Statistical Analyses.

No statistical methods were used for sample size determination; sample sizes are similar to those reported in similar studies (9, 42, 50). Datasets comprising two groups for comparison were tested for normal distribution and statistical significance using unpaired two-tailed Student’s *t* tests for parametric data, or Mann–Whitney *U* tests for nonparametric data, as detailed in figure legends. Datasets comprising more than two groups for comparison were tested for normal distribution and statistical significance using one-way ANOVA with Bonferroni’s multiple comparison test for parametric data, or Kruskal–Wallis with Dunn’s Multiple Comparison test for nonparametric data, as detailed in figure legends. Normality of datasets was tested using the Kolmogorov–Smirnov, Shapiro–Wilk, and D’Agostino Pearson normality test methods. Statistical analyses of percentage data were performed using nonparametric tests. Statistical analyses and graphing of data were performed using GraphPad Prism 8.

## Supplementary Material

Supplementary File

## Data Availability

All data discussed in the paper are available in the main text or *SI Appendix*.
