# Supplementary material for: Dynamic CCN3 expression in the murine CNS does not confer essential roles in myelination or remyelination
Source: Proc Natl Acad Sci U S A. 2020 Jul 10;117(30):18018–28. doi: 10.1073/pnas.1922089117 (PMC7395501; doi:10.1073/pnas.1922089117)
Supplement: Supplementary File [file pnas.1922089117.sapp.pdf]

PNAS
www.pnas.org

Supplementary Information for:

Dynamic CCN3 expression in the murine CNS does not confer essential roles in myelination or remyelination

Nira de la Vega Gallardo[1], Rosana Penalva[1], Marie Dittmer[1], Michelle Naughton[1], John Falconer[1], Jill Moffat[1], Alerie G. de la Fuente[1], José Romero Hombrebueno[2], Zhiyong Lin[3], Bernard Perbal[4], Rebecca J. Ingram[1], Emma Evergren[5] and Denise C. Fitzgerald[1]*

*Corresponding author: Denise C. Fitzgerald, d.fitzgerald@qub.ac.uk

This PDF file includes:

Figures S1 to S4
Table S1
Legends for Figures S1 to S4

www.pnas.org/cgi/doi/10.1073/pnas.1922089117

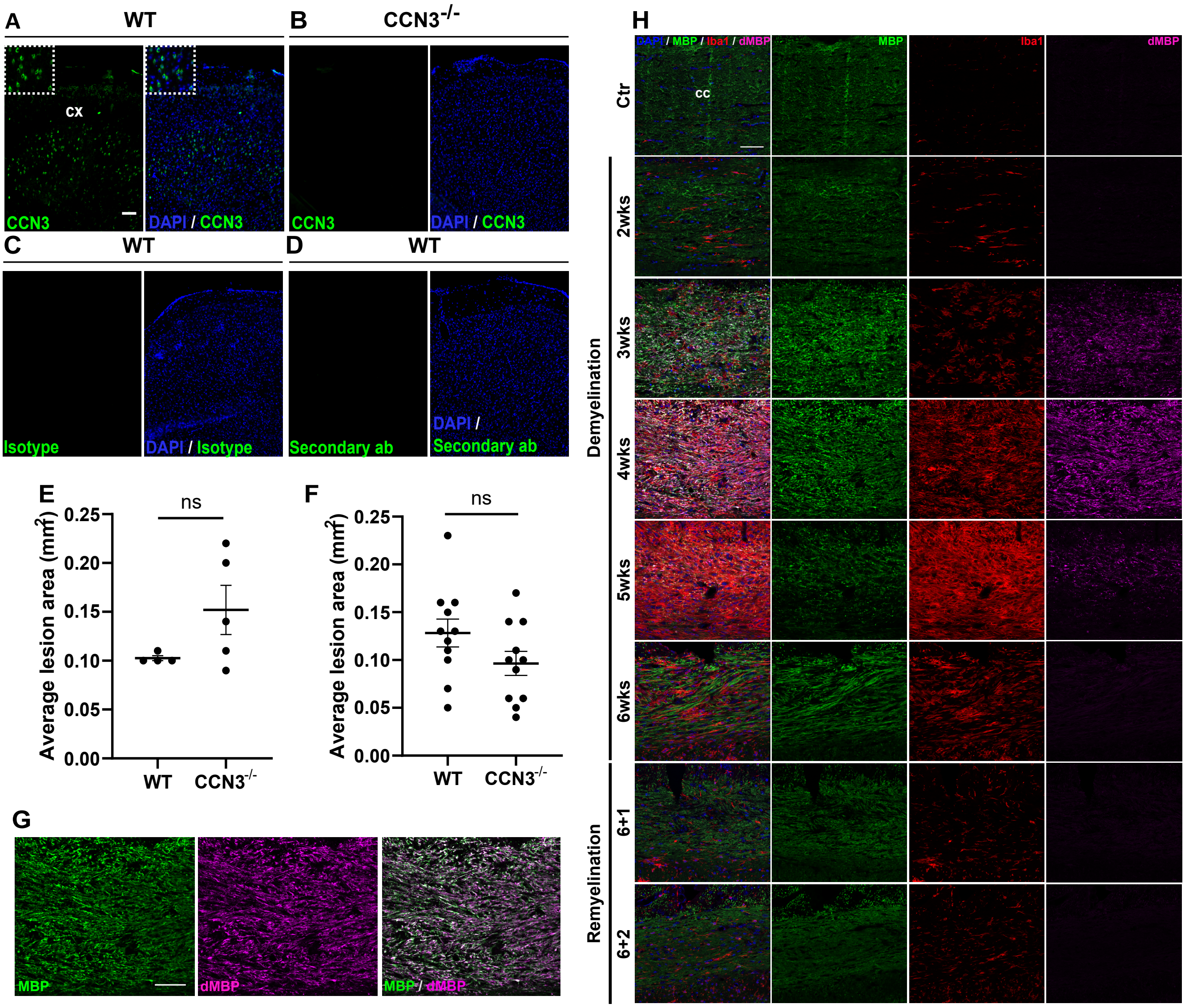

Supplementary Figure 1. Validation of staining and *in vivo* models of demyelination. (A) Staining of CCN3 in WT brain tissue. (B) Staining of CCN3 in CCN3$^{-/-}$ brain tissue. (C) Staining of isotype control of polyclonal goat IgG anti–murine CCN3 antibody in WT brain tissue. (D) Staining with secondary antibody control used to detect goat anti-murine CCN3 in WT brain tissue; no primary antibody used. cx: cerebral cortex. Quantification of average lysolecithin-induced lesion area analysed at (E) 5 dpl and (F) 14 dpl. (G) Representative confocal images of MBP and dMBP staining in the corpus callosum 4 weeks after cuprizone-induced demyelination. (H) Representative confocal images of MBP, dMBP and Iba1 staining in the corpus callosum of cuprizone-fed mice and controls. cc: corpus callosum. Scale bars: (A-D) 100 µm and (G-H) 50 µm. Data are mean ± SEM. ns: not significant. Statistical analysis: (E) Mann-Whitney U tests or (F) unpaired, two-tailed, student's t test. n = (E) 4 - 5 and (F) 11 animals per group.

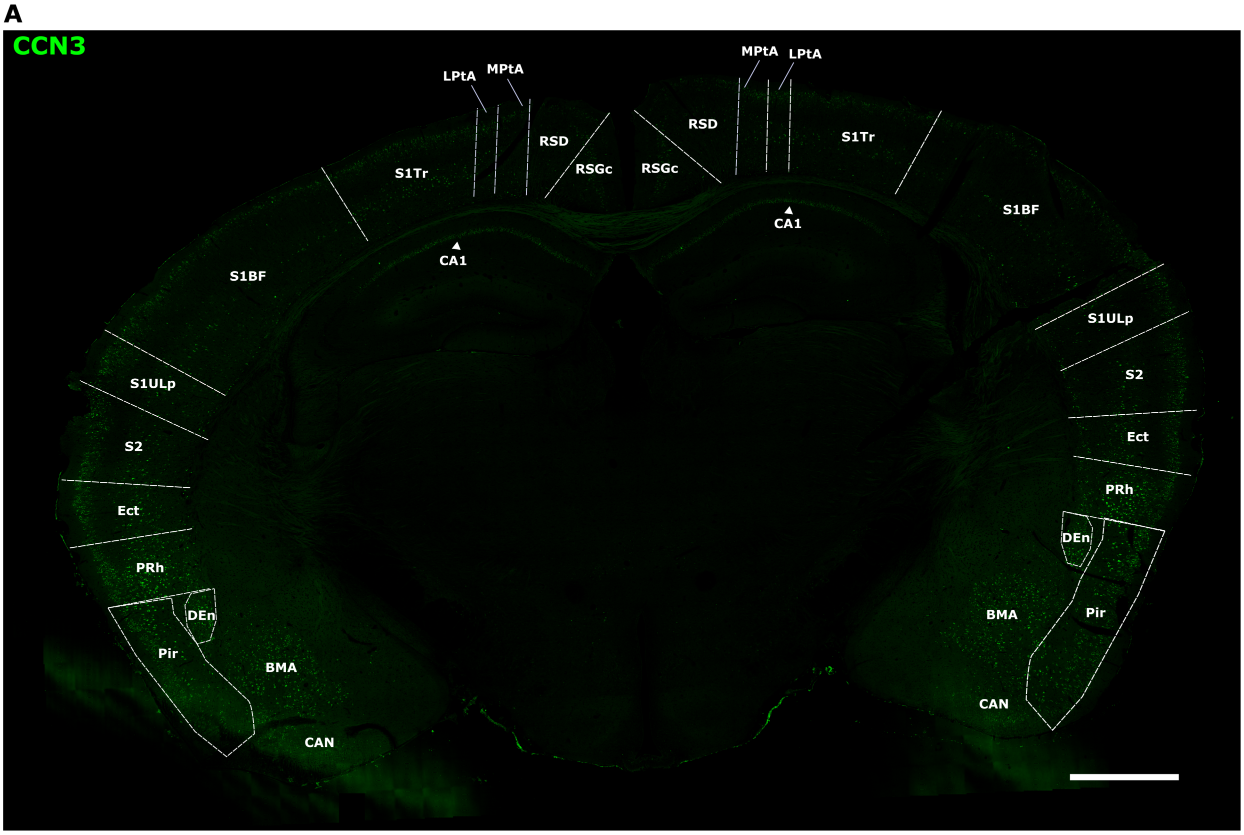

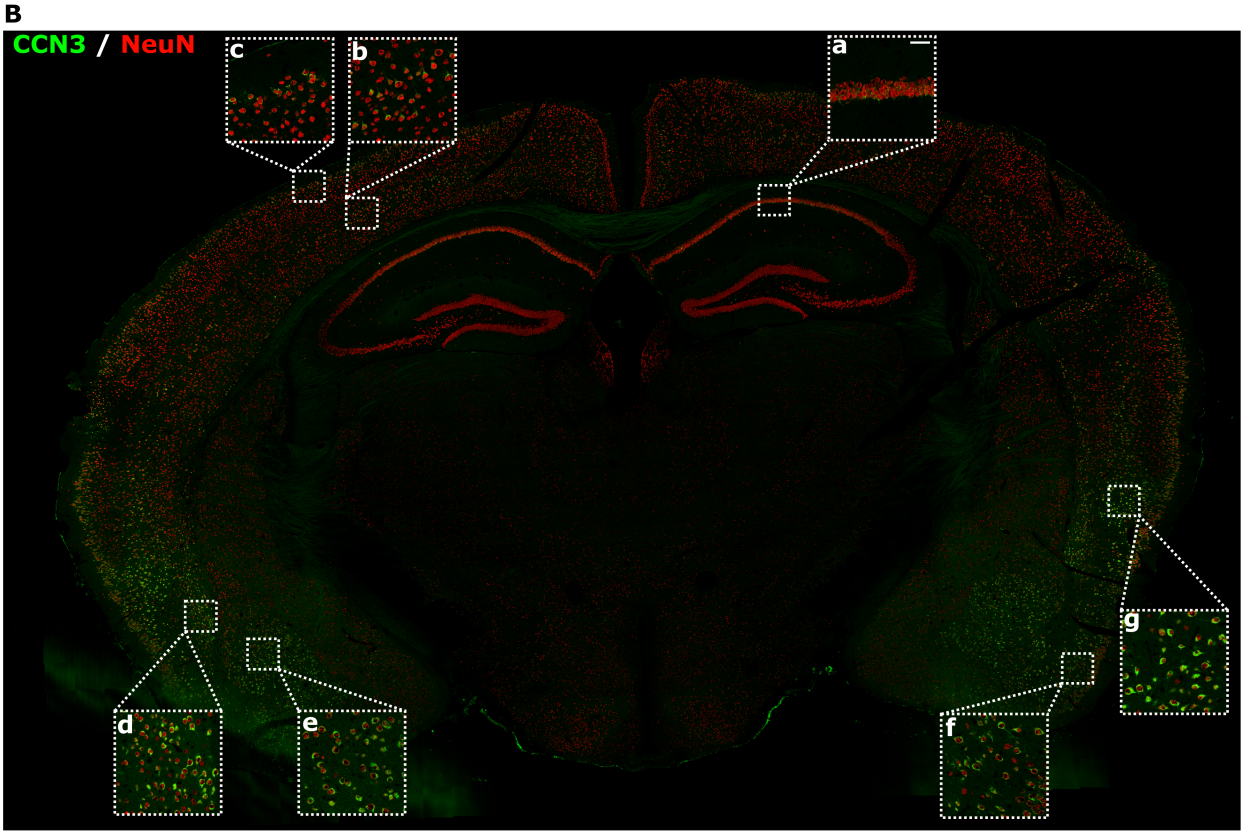

Supplementary Figure 2. CCN3 is predominantly expressed in layers 2/3 and 5 of all murine cerebral cortex anatomical regions. (A) Representative confocal stitched image of CCN3 expression in a murine coronal brain section -1.60 mm from Bregma. (B) Magnified images of CCN3 and NeuN staining in (a) hippocampal CA1 field (b) cerebral cortex layer 5 (c) cerebral cortex layer 2/3 (d) dorsal endopiriform nuclei (e) basomedial amygdala nuclei (f) piriform cortex and (g) perirhinal cortex. Scale bars are (A,B) 1 mm and (Ba-Bg) 50 µm. CAN: cortical amygdala nuclei, BMA: basomedial amygdala nuclei, Pir: piriform cortex, DEn: dorsal endopiriform nuclei, PRh: perirhinal cortex, Ect: ectorhinal cortex, S2: secondary somatosensory cortex, S1ULp: primary somatosensory cortex (upper lip), S1BF: primary somatosensory cortex (barrel field), S1Tr: primary somatosensory cortex (trunk), LPtA: lateral parietal association cortex, MPtA: medial parietal association cortex, RSD: retrosplenial dysgranular cortex, RSGc: retrosplenial granular cortex c, CA1: subfield 1 of hippocampus.

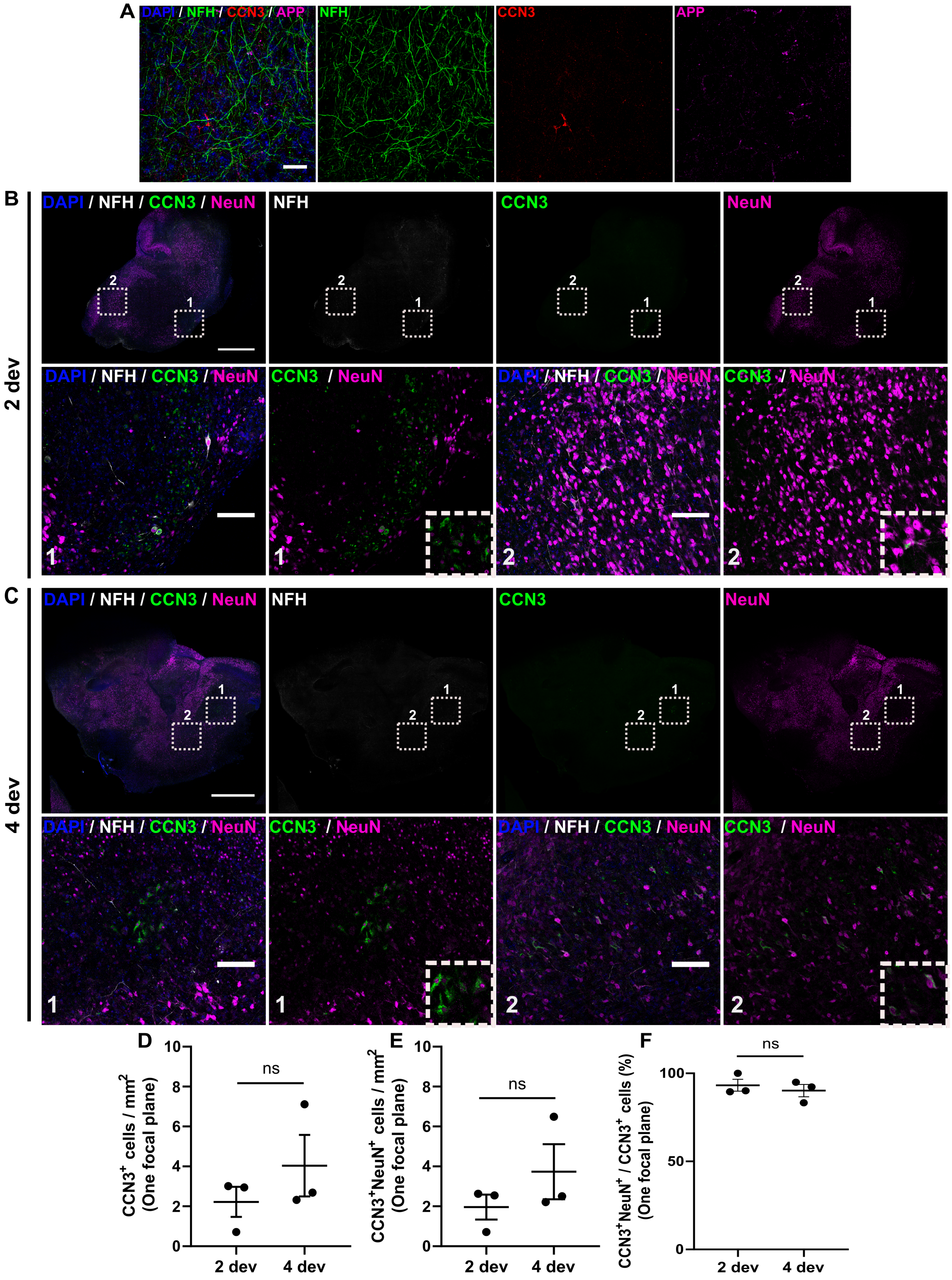

Supplementary Figure 3. CCN3 is expressed in neurons of cerebellar/brain stem slices *ex vivo*. (A) Representative confocal images of NFH, CCN3 and APP staining in brain slices at 4 days *ex vivo* (dev). Representative confocal images of NFH, CCN3 and NeuN staining in one focal plane of cerebellar/brain stem slices at (B) 2 and (C) 4 dev. (D) $CCN3^{+}$ cells, (E) $CCN3^{+}NeuN^{+}$ cells and (F) percentage of $CCN3^{+}NeuN^{+}$ cell quantification in one focal plane of 2 and 4 dev cerebellar/brain stem slices. Scale bars are (B-C) 1 mm, (B1,B2,C1,C2) 100 μm and (A) 25μm. Data are mean ± SEM. ns: not significant. Statistical analysis: (D,F) Mann-Whitney U tests or (E) unpaired, two-tailed, student's t test. n = 3 animals per group.

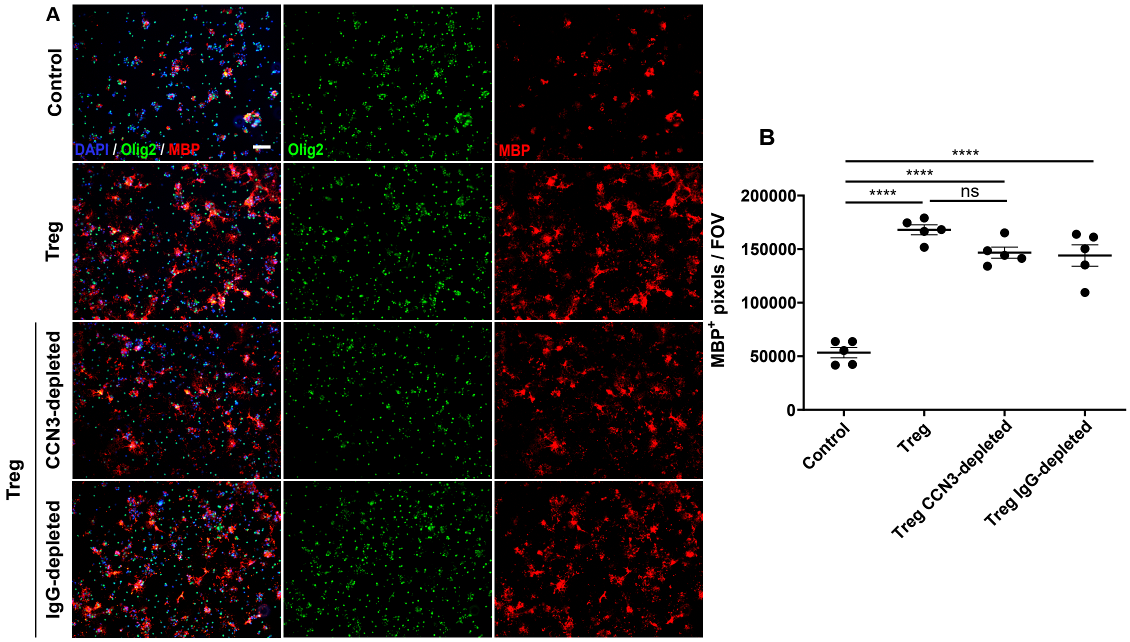

Supplementary Figure 4. CCN3 does not directly promote OPC differentiation *in vitro*. (A) Representative images of Olig2 and MBP staining in pure OPC cultures treated with control media, Treg-conditioned medium (CM), CCN3-depleted Treg-CM and isotype control-treated Treg-CM. Scale bar: 100 µm. (B) MBP^+ pixels / field of view (FOV) quantification in pure OPC cultures treated as described above. Data are mean ± SEM. ns: not significant, ****$p < 0.0001$. Statistical analysis: one-way ANOVA with Bonferroni's multiple comparison test. Data representative of 2 experiments. n = 5 wells per group.

Supplementary Table 1. Antibodies used for immunofluorescence staining of CNS frozen sections.					
**Antibody**	**Primary / Secondary**	**Dilution**	**Clone**	**Supplier**	**Catalogue number**
goat anti-mouse CCN3 (reconstituted at 0.2mg/ml)	Primary	1:100	Polyclonal	R&D systems	AF1976
rabbit anti-mouse Olig2	Primary	1:200	Polyclonal	Millipore	AB9610
mouse anti-mouse APC	Primary	1:100	CC1	Abcam	ab16794
rat anti-mouse Ki67	Primary	1:500	SolA15	eBioscience	14-5698-82
mouse anti-mouse NF200	Primary	1:400	N52	Millipore	MAB5266
chicken anti-mouse NFH	Primary	1:250 -1:400	Polyclonal	EncorBiotech	CPCA-NF-H
rabbit anti-mouse NeuN	Primary	1:500	EPR12763	Abcam	ab177487
rat anti-mouse MBP	Primary	1:600	12	Millipore	MAB386
rabbit anti-mouse dMBP	Primary	1:500	Polyclonal	Sigma	AB5864
rabbit anti-mouse Iba1	Primary	1:800	Polyclonal	Wako	019-19741
rabbit anti-mouse APP	Primary	1:300	Y188	Abcam	ab32136
chicken anti-GFAP	Primary	1:1000	Polyclonal	Abcam	ab4674
donkey anti-goat AF488	Secondary	1:200	Polyclonal	ThermoFisher Scientific	A-11055
donkey anti-rabbit AF568	Secondary	1:200	Polyclonal	ThermoFisher Scientific	A-10042
donkey anti-rabbit AF594	Secondary	1:200	Polyclonal	ThermoFisher Scientific	A-21207
donkey anti-mouse AF594	Secondary	1:200	Polyclonal	ThermoFisher Scientific	A-21203
donkey anti-chicken FITC	Secondary	1:500	Polyclonal	ThermoFisher Scientific	SA1-72000
goat anti-chicken AF488	Secondary	1:200	Polyclonal	ThermoFisher Scientific	A-11039
goat anti-rabbit AF488	Secondary	1:200	Polyclonal	ThermoFisher Scientific	A-11008
goat anti-mouse AF647	Secondary	1:200	Polyclonal	ThermoFisher Scientific	A-21236
goat anti-mouse AF594	Secondary	1:200	Polyclonal	ThermoFisher Scientific	A-11032
goat anti-mouse AF568	Secondary	1:200	Polyclonal	ThermoFisher Scientific	A-11004
goat anti-rat AF594	Secondary	1:200	Polyclonal	ThermoFisher Scientific	A-11007
